# Microwave Drying of Tricholoma Matsutake: Dielectric Properties, Mechanism, and Process Optimization

**DOI:** 10.3390/foods14173054

**Published:** 2025-08-29

**Authors:** Siyu Gong, Yifan Niu, Chao Yuwen, Bingguo Liu

**Affiliations:** 1 School of Metallurgical and Energy Engineering, Kunming University of Science and Technology, Kunming 650093, China; 20213102005@stu.kust.edu.cn (S.G.); 20213102010@stu.kust.edu.cn (Y.N.); 2 Key Laboratory of Unconventional Metallurgy, Ministry of Education, School of Metallurgical and Energy Engineering, Kunming University of Science and Technology, Kunming 650093, China; 3 National Local Joint Laboratory of Engineering Application of Microwave Energy and Equipment Technology, School of Metallurgical and Energy Engineering, Kunming University of Science and Technology, Kunming 650093, China; 4 State Key Laboratory of Complex Nonferrous Metal Resources Clean Utilization, School of Metallurgical and Energy Engineering, Kunming University of Science and Technology, Kunming 650093, China

**Keywords:** tricholoma matsutake, dielectric properties, microwave drying, response surface methodology, dehydration mechanism

## Abstract

Efficient drying is crucial for the preservation and high-value utilization of tricholoma matsutake (TM). Traditional hot-air drying is inefficient, energy-intensive, and prone to quality degradation. This study investigates the application of microwave drying for TM, systematically analyzing its dielectric properties and moisture states, and elucidating the dielectric response mechanisms during drying. Response surface methodology (RSM) was employed to optimize key process parameters, including microwave power, drying time, and sample mass, and to validate the feasibility of the optimized process for industrial applications. Results revealed that the dehydration process of TM comprises three distinct stages, with free water evaporation contributing 69.8% of the total weight loss. Dielectric properties correlated strongly with apparent density and temperature, with the loss tangent (tanδ) increasing by 213.0% at higher temperatures, confirming dipole loss as the primary heating mechanism. Under optimized drying conditions (power: 620.00 W, time: 2.70 min, mass: 13.2 g), a dehydration rate (DR) of 85.41% was achieved, with a 1.50% deviation from the model-predicted values. The optimized process effectively maintained the relative integrity of the microstructure of TM, with the C/O ratio increasing from 1.03 to 1.31. Steam pressure-driven moisture migration was identified as the primary mechanism facilitating microwave-enhanced dehydration. Pilot-scale experiments scaled up the processing capacity to 15 kg/h and confirmed that the new process reduced total costs by 38% compared to traditional hot-air drying. The study developed an efficient and reliable microwave drying model, supporting industrial-scale TM processing.

## 1. Introduction

*Tricholoma* *matsutake* (TM), a rare, pine-symbiotic edible fungus, contains valuable bioactive components such as polysaccharides, proteins, and amino acids in its fruiting body [1,2]. Ethanol extracts of TM induce tumor cell apoptosis, while its polysaccharide fractions demonstrate antioxidant and immunomodulatory properties [3]. However, the fresh TM with high moisture content (87 ± 2.00%) accelerates postharvest deterioration through respiration, enzymatic browning, and microbial proliferation, leading to irreversible degradation of nutrients (e.g., polysaccharides) and diminished edibility and market value [4]. Drying is essential for extending shelf-life by reducing water activity, thereby inhibiting enzymatic activity and microbial growth, minimizing losses, and enhancing economic returns [5]. While hot-air drying is the most common technique for edible fungi [6], it suffers from low thermal efficiency (<40%) and promotes Maillard reactions under sustained high temperatures. This depletes key C_8_ aroma compounds like 1-octen-3-ol and causes cellular rupture, accelerating enzymatic oxidation [7]. The resulting loss of distinctive pine-like aromas, surface darkening, and nutritional deterioration significantly reduce commercial value [8]. Structural damage also facilitates the release of intracellular constituents that generate undesirable volatile compounds [9]. Consequently, developing rapid, low-damage dehydration technologies is critical for high-value TM processing.

**Figure 1:**
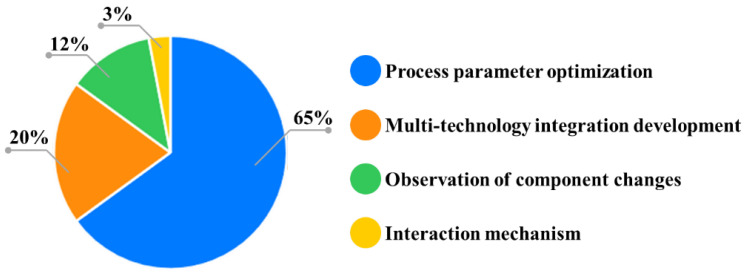
Distribution of research areas in MD of matsutake (based on literature search using keywords “microwave”, “drying”, and “matsutake”).

One promising alternative is microwave drying (MD), which offers high energy efficiency and rapid heating. MD utilizes electromagnetic waves (commonly at 2.45 GHz) to generate heat volumetrically within the material. In this process, polar molecules (chiefly water) are forced to oscillate by the high-frequency alternating field, and frictional losses from this molecular motion convert electromagnetic energy into thermal energy. This mechanism of dielectric heating produces heat simultaneously throughout the interior of the material, rather than relying on slow conduction from the surface. This synchronous internal–external heating creates vapor pressure gradients, enabling directional moisture migration and homogeneous dehydration [10]. Unlike conventional methods relying on conduction, convection, or radiation (exterior to interior), microwave energy directly activates dielectric-sensitive substances at the molecular level. The congruent heat and mass transfer directions can reduce drying time to 1/10–1/100 of traditional methods, minimizing thermal exposure and facilitating rapid dehydration at lower bulk temperatures, thereby preserving quality [11]. Although dielectric heterogeneity can cause localized overheating, microwave volumetric heating (typically 2.45 GHz) generates internal heat sources, ensuring more uniform energy distribution than gradient-driven processes and mitigating surface hardening and internal drying hysteresis [12,13]. As Table 1 and Figure 1 summarize, MD outperforms hot-air, freeze, and vacuum drying in dehydration rate (DR), nutrient retention, product quality, and microbial safety. Additionally, non-thermal microwave effects exhibit bactericidal properties by dielectric breakdown of microbial membranes [14]. Modular systems with tunable power density enable precise control, and parameter optimization (e.g., intermittent irradiation, 45–60 °C surface limits) can maintain uniform moisture distribution while preserving compounds like tricholomic acid [15]. These findings underscore the potential of MD to improve both the efficiency and the quality of drying for various food materials.

However, it is important to note that microwave heating is not without challenges. Variations in dielectric properties within a material (dielectric heterogeneity) can cause uneven absorption of microwave energy, potentially leading to localized overheating or “hot spots”. When microwaves act on food, the ability of the food to absorb electromagnetic wave energy and generate heat mainly depends on the dielectric properties of the food, which are usually represented by the relative permittivity ( ${\varepsilon_{r}}^{\prime }$) and the relative dielectric loss ( ${\varepsilon_{r}}^{\prime \prime }$). ${\varepsilon_{r}}^{\prime }$ reflects the ability to store static electric energy, while ${\varepsilon_{r}}^{\prime \prime }$ reflects the ability to convert static electric energy into thermal energy. Food with good dielectric properties typically has excellent microwave absorption performance. Jiang et al. [22] used microwave blanching to increase the dielectric factor of agaricus bisporus slices and thus reduced the microwave vacuum drying duration. Similarly, Wang et al. [23] also found that the microwave freeze-drying rate was significantly higher after adding salt and sugar, because adding both salt and sugar can enhance the dielectric properties of potatoes. Therefore, determining the dielectric properties of TM is crucial for optimizing the microwave drying rate and uniformity.

In addition, optimizing MD parameters (microwave power, drying time, material loading) for fresh TM is crucial to enhance storage stability, quality, and commercial value. Response surface methodology (RSM) is a powerful tool for optimizing these parameters through mathematical modeling and experimental design. It uses polynomial equations and 3D surface plots to predict responses and visualize interactions and is widely applied in food engineering and chemical processes [24,25]. Previous studies demonstrate the efficacy of RSM in hybrid drying systems: Liu et al. optimized microwave–hot-air drying of purple cabbage using three variables at three levels, identifying optimal parameters (2.5 W/g microwave density, 4.0 g/g moisture transition point, 55 °C air temperature) for quality preservation [26]. Similarly, Senem Tüfekçi et al. employed Central Composite Design (CCD) to optimize sweet potato drying, achieving 61.76 min drying time with 101.97 W microwave power and 54.36 °C air temperature, while maintaining key quality indices (3.29% rehydration ratio, 36.56% water retention, 31.03% antioxidant activity retention) [27]. However, there is a lack of dedicated research on the high-temperature dielectric properties of TM and the application of RSM to optimize microwave drying parameters as well as the underlying mechanisms of microwave–TM interactions.

In conclusion, this study proposes a one-step microwave-based drying method designed to improve energy efficiency while preserving product quality. This approach aims to overcome the drawbacks associated with conventional drying techniques, which, due to prolonged exposure to high temperatures, often result in surface hardening, discoloration, and nutrient degradation. The study systematically investigates the MD process and mechanism of TM under a 2.45 GHz microwave field, with an emphasis on dielectric properties, dielectric loss mechanisms, and the microwave-enhanced dehydration behavior of TM. Furthermore, this research focuses on MD technology and systematically examines the dielectric characteristics of TM at a frequency of 2.45 GHz. The RSM is employed to optimize key drying parameters, including microwave power, drying time, and sample mass, to achieve both efficient dehydration and quality preservation. By elucidating the microwave-enhanced dehydration mechanism and analyzing microstructural and compositional changes in TM following microwave treatment, this study provides a theoretical foundation for industrial-scale drying of TM. It also validates the feasibility of MD technology from laboratory to industrial application, thereby enhancing the storage stability, nutrient retention, and commercial value of TM.

## 2. Materials and Methods

### 2.1. Materials

**Figure 2:**
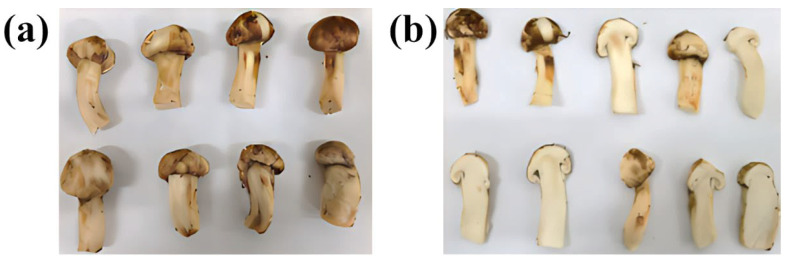
Morphological images of fresh TM in whole (**a**) and sliced (**b**) forms.

Fresh TM specimens were sourced from a designated region in Yunnan, China. Harvested samples were immediately stored in ice-packed insulated containers to preserve physicochemical integrity. Selected specimens exhibited uniform optimal coloration, size, and maturity (80–90%, via spore release analysis), with exclusion of damaged, contaminated, or infested individuals. Figure 2 shows morphological features of intact/cross-sectioned specimens.

### 2.2. Dehydration Ratio Determination in Fresh TM

Moisture content was determined via direct drying. Fresh TM samples underwent surface cleaning with a damp cloth and distilled water rinse. Residual moisture was absorbed with sterile filter paper before slicing into 2–3 mm sections. Initial mass ($m_{0}$, g) was measured using a 0.001 g precision balance. Samples were distributed evenly in trays and dried at 80 °C (±1 °C) for 24 h, with physical changes monitored every 4 h. Dried samples were cooled in a silica-gel desiccator at 25 °C before recording final mass ($m_{1}$). The procedure (preparation, drying, weighing) was triplicated with quality controls: sealed containers to prevent contamination, data-logged oven temperature monitoring, and periodic desiccant replacement. Dehydration ratio (DR, %) was calculated as follows:

(1) $$\mathrm{DR}\;=\;(\frac{m_{0}-m_{1}}{m_{0}})\;\times \;100$$

Fresh TM showed 88.47% average moisture content (Table 2), aligning with literature values (86.20%) [28] and confirming methodological validity.

### 2.3. Dielectric Property Measurement of TM

Microwave heating efficiency depends on dielectric loss characterized by relative permittivity ($\varepsilon_{r}^{\prime }$), relative dielectric loss ($\varepsilon_{r}^{\prime \prime }$), and loss tangent (tanδ). Given the high moisture content (88.47%) of TM and the elevated $\varepsilon_{r}^{\prime }$ and tanδ values of water, the microwave adsorption of TM is primarily moisturedependent. Complex permittivity $\varepsilon_{r}$ is defined as the capacity of a material to absorb and store microwave radiation and was calculated using the following equation:

(2) $$\varepsilon_{r}=\varepsilon_{r}^{\prime }-j\varepsilon_{r}^{\prime \prime }$$

where j is an imaginary unit, *j^2^*= $\sqrt{-1}$; $\varepsilon^{\prime }$ is the real part of the complex permittivity, indicating the ability of the energy transferred from the external electric field to be stored in the material; and $\varepsilon_{r}^{\prime \prime }$ is the imaginary part, representing the ability of the dielectric to convert microwave energy into heat. The $\varepsilon_{r}$ quantifies the microwave energy storage ($\varepsilon^{\prime }$) and conversion to heat ($\varepsilon^{\prime \prime }$) of a material. Both parameters are temperature- and frequency-dependent [29,30]. The dissipation efficiency is defined by the following:

(3) $$tan\;\delta =\frac{\varepsilon^{\prime \prime }}{\varepsilon^{\prime }}$$

**Figure 3:**
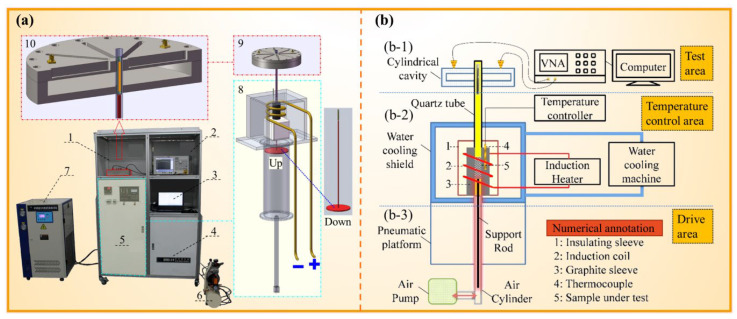
Physical picture (**a**) and testing principle diagram (**b**) of dielectric testing equipment. 1—Testing area; 2—vector network analyzer; 3—data output window; 4—transformer; 5—con trol panel, heating area, lifting control area; 6—water cooling machine; 7—air pump; 8—heating system, lifting control system; 9—test chamber; 10—test chamber.

Materials are classified as low—( tanδ < 0.1), medium—(0.1 < tanδ < 0.5), or high absorptivity ( tanδ > 0.5) based on this ratio. Dielectric properties were measured via the resonant cavity perturbation method (Figure 3), with the setup as a vector network analyzer, waveguide-coaxial adapter, coupling device, electromagnetic induction heating device, quartz sleeve, circulating water cooling system, sample lifting mechanism, cylindrical cavity, and computational software system. Samples were heated to target temperatures in an induction heater, rapidly transferred to a cylindrical cavity, and analyzed using a vector network analyzer (De Technology Co., Ltd., Beijing, China ). Frequency shifts and quality factors were converted to $\varepsilon^{\prime }$ and $\varepsilon^{\prime \prime }$ via computational software [31].

During the measure of dielectric properties, TM samples were encapsulated in quartz tubes ( Φ 0.4 × 5 cm; filled height: 4.5 cm) with high-temperature fiber to prevent oxidation and ejection. As depicted in Figure 3, samples were inductively heated to target temperatures (25–425 °C, Δ50 °C) at varied apparent densities (0.59–0.83 g/cm^3^, Δ0.04 g/cm^3^) and 2.45 GHz, then rapidly elevated into the cavity for analysis. $\varepsilon_{r}^{\prime }$, $\varepsilon^{\prime \prime }$, and tanδ were calculated from resonance frequency shifts and quality factors recorded by the analyzer, with room-temperature measurements as controls.

### 2.4. MD of TM

**Figure 4:**
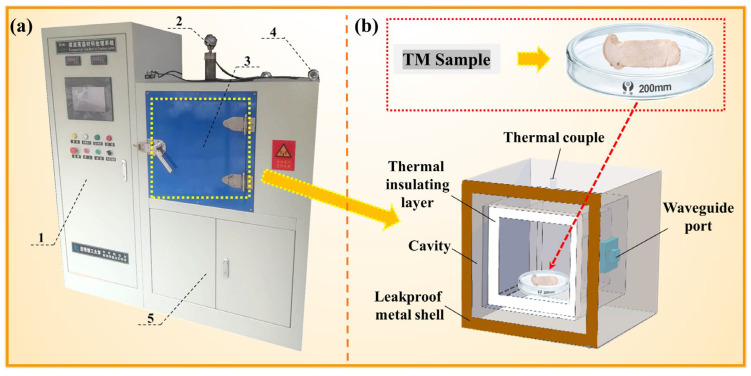
Photograph of the microwave heating equipment (**a**) and schematic diagram of its working principle (**b**): 1—control panel; 2—thermocouple; 3—microwave heating chamber; 4—thermocouple measurement port; 5—gas inlet and outlet ports.

MD was performed in a custom system (0–4.5 kW adjustable power, 2.45 GHz; Key Laboratory of Unconventional Metallurgy, Kunming University of Science and Technology; Figure 4) under argon atmosphere to prevent oxidation, which consists of magnetrons, alumina-silicate thermal insulation bricks, S-type thermocouples (WRP series, ± 4 °C accuracy), and water-cooled waveguides. Fresh TM slices were dried at varied microwave powers (300–900 W), durations (1–7 min), and masses (5–25 g). For single-factor experiments, time variation tests fixed the power at 700 W and mass at 25 g with drying times from 1 to 7 min; power variation tests used a fixed 5 min time and 25 g mass across 300–900 W; and mass variation tests maintained 700 W power and 5 min time with sample masses from 5 g to 25 g. These experimental conditions were selected based on our preliminary studies and references to previous microwave drying research [32,33]. Specifically, the chosen power range (300–900 W) and time range (1–7 min) were determined to be optimal for efficiently removing moisture from the TM slices while maintaining their structural integrity. The mass range (5–25 g) was selected to evaluate the influence of sample mass on the drying process. Post-drying, samples were desiccator-cooled and weighed (n = 5 replicates). DR data quantified parameter effects on the moisture removal.

### 2.5. Thermogravimetric (TG) and SEM-EDS Analysis

Thermal decomposition characteristics were determined using an STA 449F3 analyzer (NETZSCH, Sebul, Germany). Fresh TM samples (16.5 mg) in corundum crucibles underwent heating from 25–425 °C at 10 °C/min under 60 mL/min air flow, with mass recorded per 2 °C increment. Microstructural changes of TM samples were analyzed via TESCAN MIRA LMS (TESCKEN Trading Co., Ltd., Shanghai, China) scanning electron microscope (SEM) with a Schottky field-emission gun (15 kV, 100 pA). Imaging Resolutions: 0.9 nm (SE, 15 kV); 2.0 nm (BSE, 30 kV). Elemental distribution was determined by integrated energy-dispersive X-ray spectroscopy (EDS, Octane Pro, AMETEK, Dallas, Texas, USA). Samples (0.5 × 0.5 mm) were gold-sputtered for 30 s after mounting on SEM stubs.

### 2.6. Statistical Analysis

The analysis of variance (ANOVA) was performed using Statistical Analysis System software (SAS, version 8.1, SAS Institute Inc., Cary, NC, USA). The least significant difference (LSD) test was employed to assess the differences between means. A *p*-value of less than 0.05 was considered to indicate statistical significance.

## 3. Results and Discussion

### 3.1. Thermogravimetric Analysis of TM

**Figure 5:**
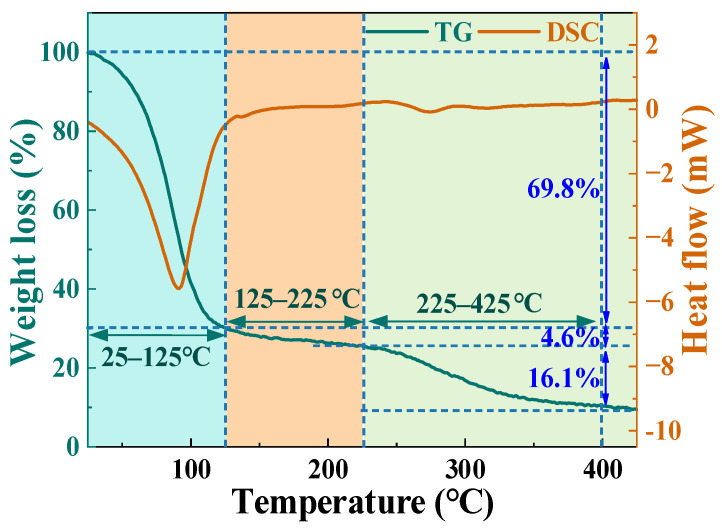
TG and DSC curves of the TM between 25 °C and 425 °C.

Thermogravimetric-differential scanning calorimetry (TG-DSC) analysis systematically revealed phase transitions and thermal effects of three water states in TM during MD (Figure 5). Under argon atmosphere (10 °C/min to 425 °C), three distinct mass loss stages occurred, as shown in Figure 5. In the first stage (25–125 °C), rapid evaporation of free water contributes to 69.8% mass loss steep TG decline and endothermic DSC peak, indicating energy absorption for hydrogen bond disruption [34]. The second stage (125–225 °C) exhibits less mobile water migration, in which 4.6% mass loss with gradual TG slope and stable DSC baseline, reflecting diffusion-limited release from microporous structures. The third stage (225–425 °C) involves the removal of bound water (16.1% mass loss) with subtle endotherm, corresponding to chemical bond cleavage and organic component decomposition. MD efficiently removes free water but risks surface cracking from rapid vaporization. While less mobile water migration minimally affects tissue integrity, its diffusion resistance limits drying efficiency. Bound water release reduces mass but degrades heat-sensitive nutrients (amino acids, vitamins) at elevated temperatures. Peak dehydration occurred at 92 °C (free water evaporation), highlighting that late-stage shrinkage impeded moisture diffusion. MD can efficiently remove free water and constitutes the main part of the drying process. However, as drying progresses, the bottleneck shifts from surface evaporation to internal diffusion, and the high-temperature phase may damage product quality. Revealing this mechanism provides a theoretical basis for balancing efficiency and quality in subsequent process optimization.

### 3.2. Dielectric Properties Analysis of TM

#### 3.2.1. Effect of Apparent Density on Dielectric Properties

**Figure 6:**
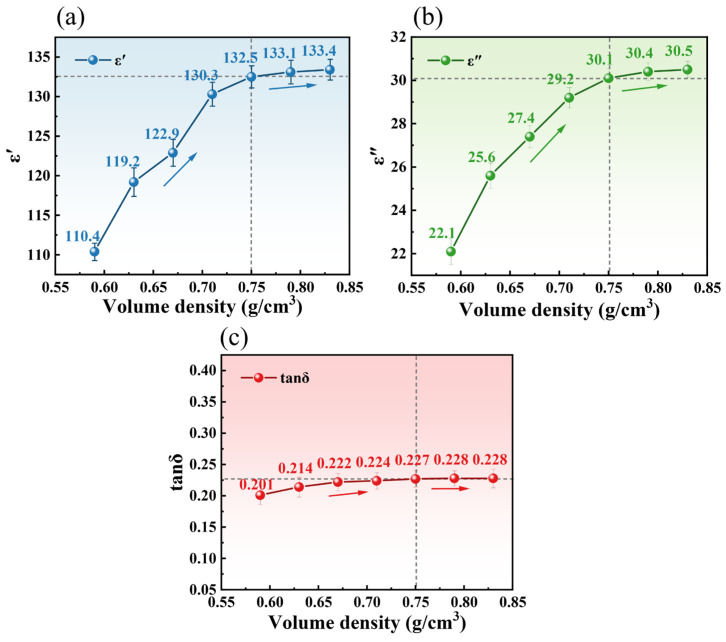
Effect of bulk density on the dielectric properties of TM at room temperature, (**a**) $\varepsilon_{r}^{\prime }$ ; (**b**) $\varepsilon_{r}^{\prime \prime }$ ; and (**c**) tanδ .

The variations in dielectric parameters ($\varepsilon_{r}^{\prime }$, $\varepsilon_{r}^{\prime \prime }$, and tanδ) of TM with apparent density (0.59–0.83 g/cm^3^) were investigated at 2.45 GHz and 25 °C, as shown in Figure 6. As the apparent density increased from 0.59 to 0.83 g/cm^3^, $\varepsilon_{r}^{\prime }$, $\varepsilon_{r}^{\prime \prime }$, and tanδ exhibited a linear upward trend, rising from 110.40, 22.10, and 0.201 to 133.4, 30.5, and 0.228, respectively. In the low-density range (0.59–0.70 g/cm^3^), weaker dielectric responses were observed, with lower values of $\varepsilon_{r}^{\prime }$, $\varepsilon_{r}^{\prime \prime }$, and tanδ. This phenomenon may be attributed to the dominance of air voids within the loosely packed structure, which reduced overall polarization capacity and energy dissipation efficiency [35]. When the apparent density increased to 0.75–0.83 g/cm^3^, reduced porosity led to structural densification, shortening intermolecular distances and enhancing dipole orientation polarization and interfacial polarization effects. Under microwave electric fields, elevated molecular density promoted synergistic oscillations between dipoles and the applied field, resulting in significant improvements in energy storage capacity and energy dissipation rate. The concurrent rise in tanδ further indicated superior electric-to-thermal energy conversion efficiency in high-density samples. Notably, when the apparent density exceeded 0.75 g/cm^3^, the growth rates of $\varepsilon_{r}^{\prime }$, $\varepsilon_{r}^{\prime \prime }$, and tanδ declined significantly. This attenuation likely originated from restricted molecular mobility caused by overcrowded packing, where enhanced intermolecular interactions counterbalanced steric hindrance effects. In conclusion, the dielectric properties of TM exhibited a strong positive correlation with apparent density. The increases in $\varepsilon_{r}^{\prime }$ and $\varepsilon_{r}^{\prime \prime }$ reflected simultaneous enhancements in energy storage and dissipation capabilities, while the rise in tanδ indicated improved energy conversion efficiency. However, excessive apparent density (>0.75 g/cm^3^) induced molecular mobility constraints, diminishing the upward trend of these parameters. This establishes an optimal apparent density range (<0.75 g/cm^3^) for maximizing microwave energy absorption and thermal conversion efficiency during TM processing.

#### 3.2.2. Effect of Temperature on Dielectric Properties

**Figure 7:**
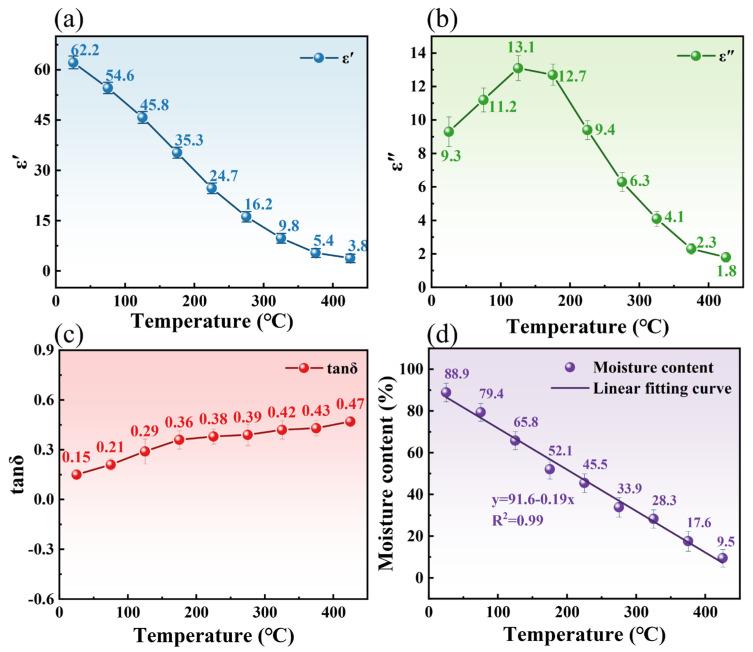
Effect of temperature on the dielectric properties of TM, (**a**) $\varepsilon_{r}^{\prime }$; (**b**) $\varepsilon_{r}^{\prime \prime }$; (**c**) tanδ; and (**d**) The fitting curve of the moisture content of TM varying with temperature..

Temperature significantly influenced the dielectric behavior of TM during microwave processing. As illustrated in Figure 7 (measured at 2.45 GHz and bulk density of 0.75 g/cm^3^), $\varepsilon_{r}^{\prime }$ value decreased from 62.20 at 25 °C to 3.80 at 425 °C, representing a 93.89% reduction, which correlated linearly with moisture content decline from 88.90% to 9.50% (Figure 7a,d). This trend aligns with established principles where water dominates dielectric polarization in biomaterials, as evidenced by Qi et al. [36] in edible fungal powders. The three-stage dehydration mechanism identified via TG-DSC (Figure 5) was as follows: free water removal (25–125 °C), weakly bound water release (125–225 °C), and bound water elimination with organic decomposition (225–425 °C) exhibited strong coupling with dielectric responses. During the free water-dominated phase (25–125 °C), $\varepsilon_{r}^{\prime }$ sharply decreased alongside a 69.80% mass loss in TG and endothermic DSC peaks, reflecting diminished dipole polarization due to rapid moisture depletion (Figure 7a). $\varepsilon_{r}^{\prime \prime }$ value displayed non-monotonic behavior, increasing from 9.30 at 25 °C to a peak of 13.10 at 125 °C (moisture content: 65.80%), then declining to 1.80 at 425 °C (Figure 7b). This peak aligns with Lombraña et al.’s [37] observation of maximum $\varepsilon_{r}^{\prime \prime }$ at intermediate moisture levels in plant tissues, attributed to the competition between the two opposite effects of ion conductivity enhancement and molecular motion limitation. The overall increase in the tanδ was 213.0% over the temperature range, as shown in Figure 7c, indicating that the electro-thermal conversion efficiency of TM continued to improve even in the high-temperature phase with extremely low moisture content [38]. The study speculated that this was related to the carbonized structure formed by thermal decomposition, which enhanced the conductive losses. Beneroso et al. [39] reported analogous behavior in pyrolyzed biowastes, where carbonaceous phases induced conduction losses above 400 °C, consistent with the tanδ rise observed here at 425 °C. The dielectric response of the material is highly coupled with the three-stage dehydration process revealed by TG-DSC, confirming that the state of moisture is the key determinant of heating efficiency.

#### 3.2.3. Dielectric Loss Mechanism of TM

**Figure 8:**
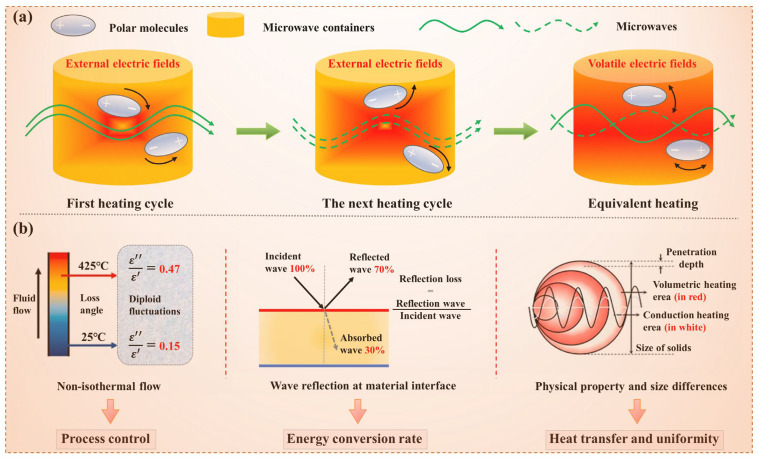
Main dielectric loss mechanism of TM, (**a**) The motion mechanism of polar molecules in microwave field; (**b**) The change mechanism of dielectric properties of TM in microwave field.

Electrically neutral atoms and molecules, harboring separate positive and negative charges, exhibit external polarity. TM would be polarized under an external electric field, exhibiting a dielectric response. This polarization, driven by dipoles, is where the thermal effect of substances in microwave fields originates. Dielectric materials, with their charge carriers realigning under an electric field, compensate for the field by moving charges in opposite directions. At microwave frequencies, dipole orientation polarization resulting in losses. The $\varepsilon_{r}^{\prime \prime }$ value of TM decreases from 9.3 to 1.8 as the temperature increases from 25 °C to 425 °C, indicating that dipole loss was the primary heating mechanism. Specifically, microwave irradiation polarized TM molecules, generating dipoles that absorb microwave energy, converting it uniformly into heat, as Figure 8a demonstrates. The reduction in dipole loss is primarily attributed to the increased temperature, which leads to a significant outflow of polar water molecules from the TM, reducing the total number of polar molecules and thus lowering the dipole loss. Moreover, material-specific dielectric properties, influenced by structure and composition, lead to varied responses. As shown in Figure 8b, the tanδ of TM increases from 0.15 at 25 °C to 0.47 at 425 °C, qualifying it as a robust microwave absorber. At high temperatures, TM may convert into materials with specific carbon structures, which could exhibit some degree of electrical conductivity, leading to conduction losses. These findings highlight the industrial potential of microwave-dried TM.

### 3.3. Influencing Factors in MD of TM

**Figure 9:**
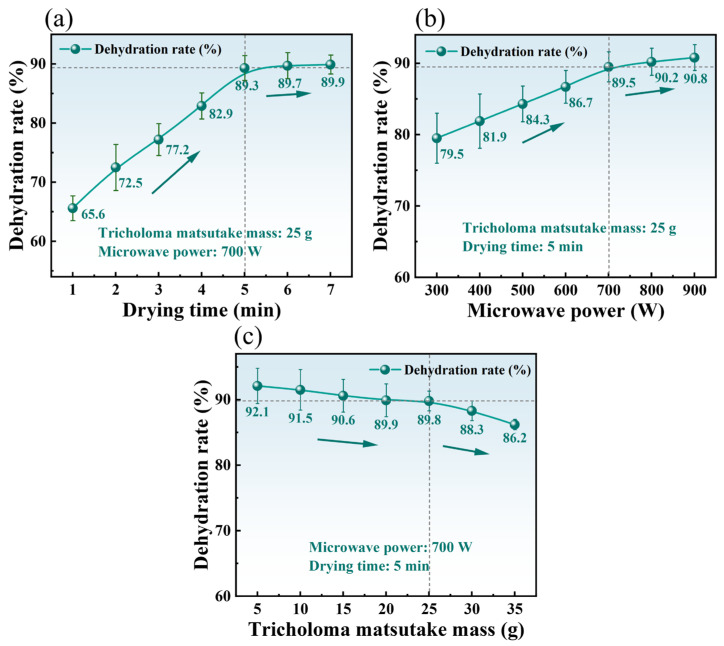
Single-factor experiments of TM MD. (**a**) Effect of drying time on DR; (**b**) effect of microwave power on DR; and (**c**) effect of microwave power on DR.

The DR of TM during MD is governed by several key process parameters, including drying time (1–7 min), microwave power (300–900 W), and sample mass (5–35 g). Single-factor experiments were conducted to quantify the individual impacts of these variables on dehydration kinetics, with results presented in Figure 9. The detailed analysis is as follows.

#### 3.3.1. Effect of Drying Time on DR

Figure 9a shows the effect of drying time on the DR under fixed microwave power (700 W) and sample mass (25 g). The DR increased progressively from 65.60% to 89.90% as drying time extended. A particularly sharp rise in DR (Δ23.70%) occurred within the first 5 min, likely attributable to the rapid evaporation of readily available free water. Further drying time extension (5 to 7 min) resulted in significantly smaller increments in DR (Δ0.60%), suggesting higher energy requirements, potentially associated with the liberation of bound water from cellular matrices. These trends align with observations by Giri et al. [40] for microwave-dried mushrooms, who also reported an initial rapid dehydration phase driven by free water evaporation, followed by progressively slower rates. This pattern reflects the dynamic nature of MD: high initial DR facilitated by rapid evaporation, succeeded by lower rates constrained by moisture diffusion limitations.

#### 3.3.2. Effect of Microwave Power on DR

Microwave power is a critical parameter influencing dielectric drying. Figure 9b illustrates the DR of TM under varying microwave power (300–900 W) at constant sample mass (25 g) and drying time (5 min). The DR increased from 79.50% to 90.80% with increasing power. This enhancement is attributed to the intensified electromagnetic field, which elevates absorbed energy density and surface temperature proportionally, thereby accelerating moisture evaporation kinetics. The incremental gain in DR diminished beyond 700 W (Δ10% increase from 300 to 700 W vs. Δ1.3% increase from 700 to 900 W). This suggests that the dehydration process transitions from being dominated by surface evaporation to being limited by internal diffusion. This inflection likely arises from rapid surface moisture depletion establishing steep moisture concentration gradients; internal moisture migration through the cellular matrix then becomes the rate-limiting step, requiring more time. This observation is consistent with Zhou et al.’s findings on microwave processing of Quercus variabilis [41], who documented similar non-linear drying rate patterns where maximum DR increased with power but exhibited declining marginal gains beyond critical thresholds [42]. Therefore, considering the industrial application and economic benefits, 700 W was selected as the optimal microwave power.

#### 3.3.3. Effect of Sample Mass on DR

Figure 9c demonstrates the mass-dependent DR of TM under fixed microwave parameters (700 W, 5 min). An inverse correlation was observed: DR decreased from 92.10% to 86.20% as sample mass increased from 5 g to 35 g. This decline is primarily attributed to microwave energy distribution effects. Increasing the mass raises the total moisture content requiring evaporation while reducing the microwave energy density per unit mass. Consequently, the vapor pressure differential and moisture gradient between the sample interior and surroundings are reduced. This attenuates the driving force for internal moisture migration, leading to slower moisture diffusion and potentially longer drying times [43]. Additionally, increased mass extends the moisture diffusion pathways and resistance, further lowering the DR. This observation is consistent with previous studies on mass effects in MD. For instance, Zhang et al. demonstrated that increased sample mass reduces the drying rate per unit mass [44]. These findings indicate that controlling sample mass is critical for optimizing DR in practical drying processes.

In summary, increasing both drying time and microwave power generally enhanced the DR of TM, whereas greater sample mass reduced it. These trends are governed by microwave energy distribution and moisture evaporation/diffusion dynamics. The initially high DR stems from rapid free water evaporation, while the subsequent slowdown is constrained by the removal of bound water and internal moisture diffusion limitations.

### 3.4. RSM for Optimizing MD of Fresh TM

Based on the single-factor experiments, this study employed RSM with a CCD and a multiple quadratic regression model to analyze the interactive effects of microwave power (*A*), drying time (*B*), and sample mass (*C*) on the DR of TM. A statistically reliable optimization model was established by Design-Expert software (Version 10, Stat-Ease Inc., Minneapolis, MN, USA). Following experimental design and data analysis, the impacts of various process parameters on the DR were determined.

#### 3.4.1. Model Construction and Regression Analysis

The model utilized the DR as the response variable. Microwave power (*A*), drying time (*B*), and mass (*C*) were selected as independent variables. A three-factor, five-level CCD experimental design was implemented, comprising 20 runs (including factorial points, axial points, and center points) to evaluate the quadratic effects and interactions of the variables. The coded values and operational ranges for each factor are defined in Table 3. Regression analysis was applied to fit a quadratic model to the experimental data. The adequacy of the model was assessed based on statistical criteria. A model was considered statistically significant (*p*-value < 0.05) and well-fitted when the coefficient of determination ($R^{2}$) exceeded 0.8 [45]. Such a validated model can provide practical guidance for optimizing TM drying. The experimental design matrix (based on CCD) and the corresponding experimental results for MD of TM are presented in Table 4.

The mathematical relationship between the independent variables (e.g., microwave power (*A*), drying time (*B*), and material loading (*C*)) and the DR was modeled using a second-order polynomial equation:

(4) $$DR=84.38+11.03A+4.37B-3.41C-5.31A^{2}-1.89B^{2}$$

The sign and size of each coefficient indicate the direction and relative strength of the influence of the factor on the linear, interaction, and quadratic terms of DR. Equation (4) shows a positive linear effect for microwave power (*A*) and drying time (*B*) and a negative linear effect for sample mass (*C*). This suggests that, within the studied range, increasing microwave power or drying time increases DR, while increasing sample mass decreases it. The interaction and quadratic terms further refine this relationship. The adequacy of the quadratic model was rigorously evaluated using ANOVA [46,47]. Key statistical parameters are summarized in Table 5.

The model was highly statistically significant (F-value = 44.73, *p*-value < 0.0001), indicating it reliably explains the variation in DR. In this case, the terms A, B, C, $A^{2}$, and $B^{2}$ were identified as significant contributors to the model. The fitted $R^{2}$ was 0.95, with an ${R_{adj}}^{2}$ of 0.95 and a low C.V. % (3.19, <15.00), further confirming that the model adequately described the experimental data. Additionally, the predicted $R^{2}$ value of 0.84 was in reasonable agreement with the ${R_{adj}}^{2}$ value of 0.95, with a difference of less than 0.20 between the two. The signal-to-noise ratio (*Adeq Precision*) of 22.84, significantly greater than 4.00, also indicated a strong model signal. These findings suggest that the model is appropriate for exploring the design space. Moreover, the lack-of-fit test was nonsignificant (*p*-value = 0.0590) [48], further supporting the consistency between experimental observations and theoretical predictions. Collectively, these statistical metrics confirm the quadratic model (Equation (4)) is robust, reliable, and suitable for simulating and optimizing the MD process of fresh TM within the defined experimental domain.

**Figure 10:**
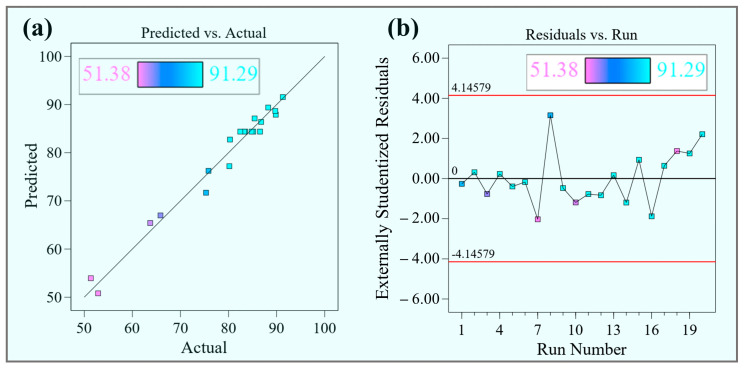
(**a**) Predicted vs. actual DR values of the model; (**b**) residual analysis.

Figure 10 presents the experimental versus predicted DR values from the response surface experiments. The scatter plot (Figure 10a) shows data points closely distributed along the 45° line, indicating good agreement between the observed experimental results and the values predicted by the regression model (Equation (4)). Furthermore, the residual plot (Figure 10b) displays a random distribution of residuals with no discernible patterns, suggesting the absence of systematic error and supporting the model’s homoscedasticity. These graphical analyses collectively confirm the reliability of the quadratic regression model for simulating the MD behavior of TM [49]. The validated model provides a robust scientific basis for process optimization studies.

#### 3.4.2. Response Surface Analysis and Process Optimization

Based on the significant terms identified in the ANOVA of the quadratic model, microwave power (*A*) exerted the strongest influence on the DR of TM, while drying time (*B*) and sample mass (*C*) had comparatively weaker, though statistically significant, effects. To elucidate the complex interactions between factors and identify optimal conditions, response surface analysis was performed using contour plots and 3D surface plots. A target DR range of 80–85% was defined as the optimal drying endpoint.

**Figure 11:**
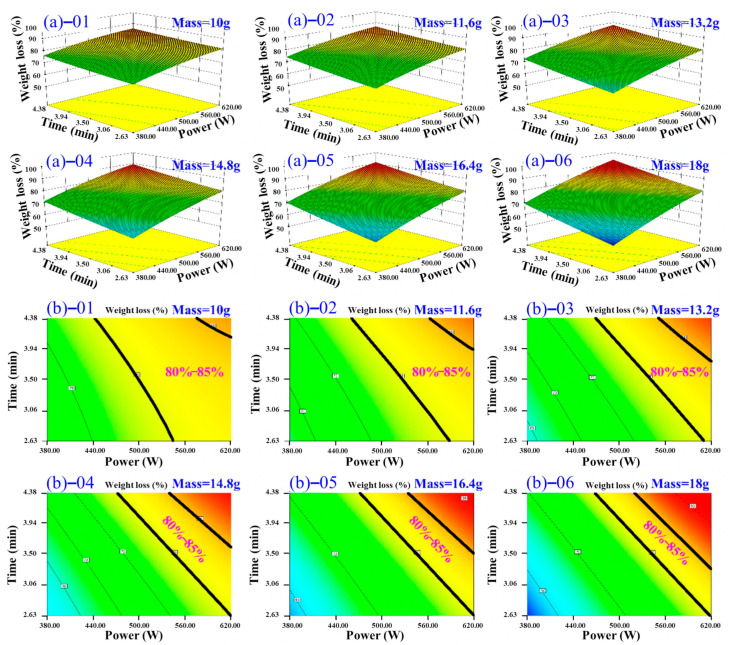
Three-dimensional response surfaces illustrating TM DR variations under fixed microwave power and time across different material mass, (**a**)–01 10 g; (**a**)–02 11.6 g; (**a**)–03 13.2 g; (**a**)–04 14.8 g; (**a**)–05 16.4 g; and (**a**)–06 18 g; and contour plots illustrating TM DR variations under fixed microwave power and time across different material mass, (**b**)–01 10 g; (**b**)–02 11.6 g; (**b**)–03 13.2 g; (**b**)–04 14.8 g; (**b**)–05 16.4 g; and (**b**)–06 18 g.

Figure 11 illustrates the interactive effects of processing time and microwave power on the DR of TM under different material mass. As the material mass increases from 10 g to 18 g, the power required to achieve target DR rises significantly (450.00 W→620.00 W), while the drying time remains stable within the range of 2.0–4.0 min. Concurrently, the attainable dehydration range broadens from 75–85% (Figure 11b-01) to 60–85% (Figure 11b-05,06). This expansion is primarily attributed to partial dehydration inefficiencies occurring under low-power or short-duration conditions at higher material loads. Moreover, the operational window for achieving optimal dehydration (80.0–85.0%) progressively narrows by 52.0% and shifts towards higher power levels (>580.00 W) and longer processing times (>2.5 min), necessitating stricter process control for larger batch sizes. This observed behavior aligns with fundamental microwave heating principles: an increase in material quantity elevates the thermal energy demand per unit time, thereby requiring proportional amplification of microwave power to maintain uniform heating and overall kinetics for moisture removal. These findings were consistent with the study by Teleken et al. [50]. Notably, even at the maximum material loading tested (18.0 g), higher microwave power levels such as 620.0 W enable efficient TM drying within a short time (2.63 min). This underscores the critical role of microwave power intensity in ensuring both the efficiency and effectiveness of the drying process, particularly when adapting to varying processing volumes.

**Figure 12:**
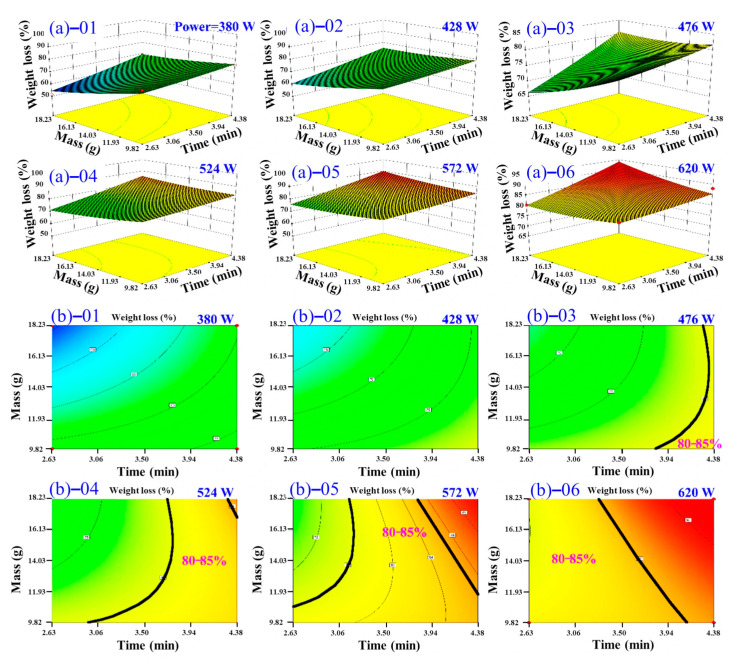
Three-dimensional response surfaces illustrating TM DR variations under fixed material mass and time parameters across variable microwave power, (**a**)–01 380 W; (**a**)–02 428 W; (**a**)–03 476 W; (**a**)–04 524 W; (**a**)–05 572 W; and (**a**)–06 620 W; and contour plots illustrating TM DR variations under fixed material mass and time parameters across variable microwave power, (**b**)–01 380 W; (**b**)–02 428 W; (**b**)–03 476 W; (**b**)–04 524 W; (**b**)–05 572 W; and (**b**)–06 620 W.

Figure 12 illustrates the interactive effects of material mass and processing time on the DR of TM under varying microwave power levels. As depicted in Figure 12a, dehydration proceeds relatively slowly at 380.00 W but accelerates significantly at 620.00 W, confirming microwave power as the dominant factor governing moisture removal efficiency. Within fixed power conditions, smaller TM masses attain target DR faster, whereas increased mass necessitates longer durations to achieve equivalent DR levels or yields lower DR within identical processing times. Further analysis of the 80–85% dehydration range in Figure 12b reveals that elevated power substantially reduces the time required to reach this critical moisture threshold. For instance, achieving target dehydration requires at least 4.38 min at 380.00 W but only 2.63 min at 620.00 W, representing a 40% reduction in processing time. Higher power levels more reliably ensure optimal dehydration outcomes for a given mass. This behavior originates from microwave power directly governing energy input per unit time: higher power intensifies heating rates, amplifies molecular agitation and phase transitions, and enhances moisture diffusivity, consistent with thermodynamic and mass transfer principles. These observations align with studies such as Zheng et al. [51], where increased thermal energy input (analogous to microwave power elevation) improved dehydration efficacy. Consequently, optimizing microwave power emerges as the most direct strategy to accelerate drying kinetics, ensure consistent product quality, and broaden operable mass ranges for industrial-scale processing.

**Figure 13:**
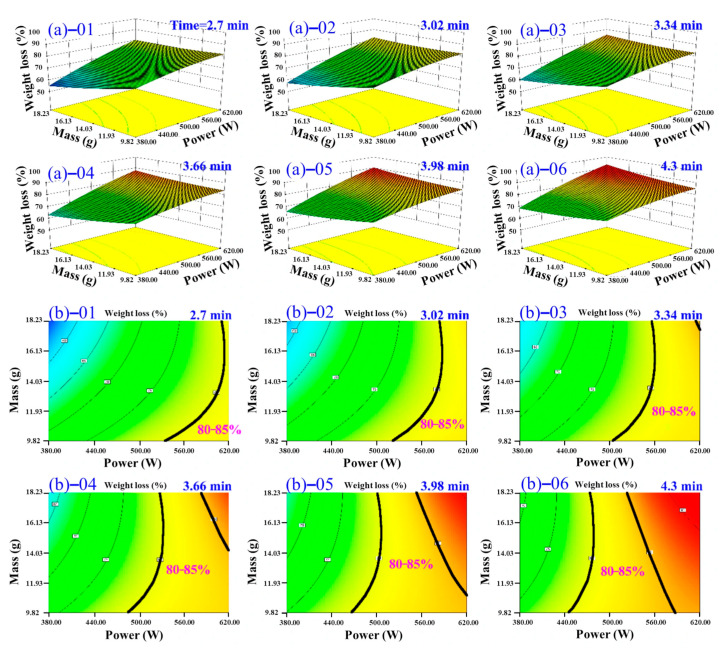
Three-dimensional response surfaces illustrating TM DR variations under fixed material mass and microwave power parameters across variable time, (**a**)–01 2.7 min; (**a**)–02 3.02 min; (**a**)–03 3.34 min; (**a**)–04 3.66 min; (**a**)–05 3.98 min; and (**a**)–06 4.3 min; and contour plots illustrating TM DR variations fixed material mass and microwave power parameters across variable time, (**b**)–01 2.7 min; (**b**)–02 3.02 min; (**b**)–03 3.34 min; (**b**)–04 3.66 min; (**b**)–05 3.98 min; and (**b**)–06 4.3 min.

Figure 13 illustrates the combined influence of material mass and microwave power on the DR of TM across varying drying durations. The data demonstrate that, for fixed mass and power conditions, the DR of TM progressively increases with extended drying time. For instance, DR values presented in Figure 13a-01 (shorter time, e.g., 2.70 min) were consistently lower than those achieved in Figure 13a-06 (longer time, e.g., 4.30 min), indicating that prolonged microwave exposure enhances moisture removal efficacy under constant operating parameters. Analysis of the 80–85% DR range (Figure 13b) reveals that diverse mass–power combinations can achieve target dehydration within different fixed drying times. Increasing drying time initially expands the feasible mass–power region. However, excessive extension beyond the optimal time required for 80–85% DR leads to over-drying, causing the effective operational region to contract due to product quality degradation. This highlights a critical trade-off: while moderately increasing drying time enhances dehydration, prolongation exceeding the necessary threshold induces over-drying. This compromises product integrity through risks including pyrolytic damage (structural carbonization), nutrient degradation, and deterioration of organoleptic properties, ultimately diminishing commercial value. Therefore, precise temporal control is paramount for maintaining quality standards and economic viability. These observations align with Teleken et al. [50], who reported a plateau in DR enhancement beyond specific time thresholds accompanied by elevated quality deterioration risks.

#### 3.4.3. Verification Experiments of the Optimization Model

**Figure 14:**
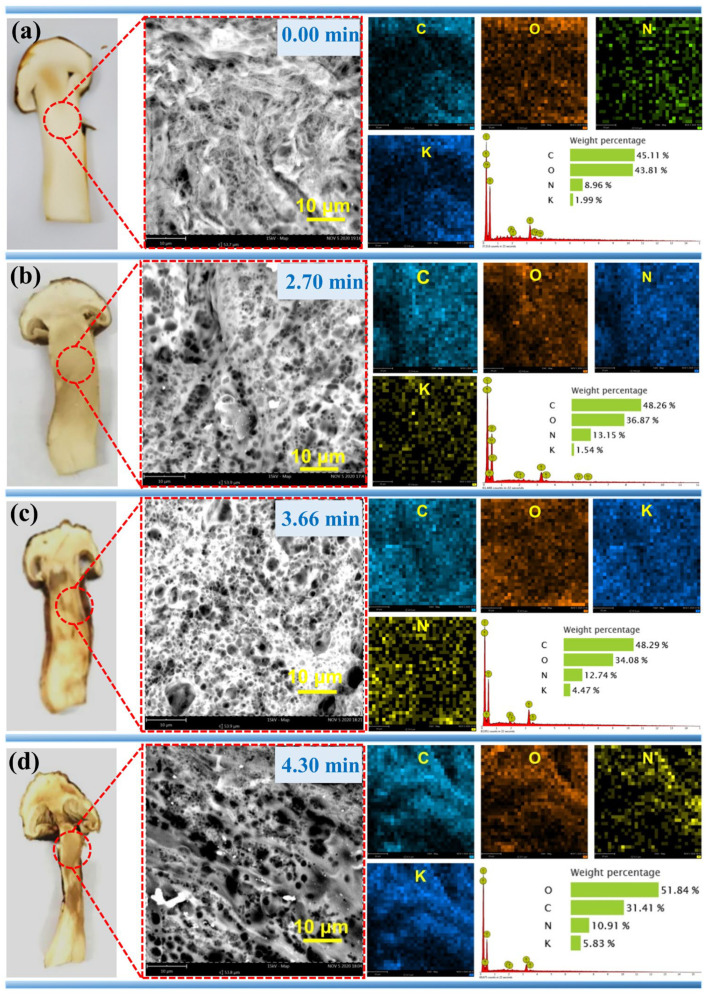
Impact of MD time on TM specimen characteristics: macroscopic morphology, SEM microstructure, and EDS compositional profiles under fixed parameters (Power: 620.00 W; Mass: 13.2 g). (**a**) 0.00 min, (**b**) 2.70 min, (**c**) 3.66 min, and (**d**) 4.30 min.

To validate the quadratic regression model, verification experiments were conducted under the optimized conditions (microwave power: 620.00 W; drying time: 2.70 min; material mass: 13.20 g). Moreover, 13.20 g TM samples were also experimented at 620.00 W for different drying time like 0.00 min, 3.66 min, and 4.30 min, to prevent over-drying. The parallel experiments under the three conditions were all repeated three times to obtain average DR values (Table 6). The relative deviation (1.50%) falls well within the standard deviation of model (2.40%), confirming the reliability of the response surface model predictions and process optimization. Additionally, SEM-EDS analysis was conducted to evaluate the impact of varying drying times (0.00, 2.70, 3.66, and 4.30 min) on the surface morphology, internal microstructure, and cellular composition of TM under optimized parameters (microwave power: 620.00 W; mass: 13.20 g). The results are presented in Figure 14.

Figure 14 compares the SEM-EDS spectra of TM samples treated with different MD times (0.00, 2.70, 3.66, and 4.30 min). The fresh sample (Figure 14a) displays uniformly distributed cellular structures with intact cell walls and a C/O mass ratio of 1.03. after 2.70 min of drying (Figure 14b), structural contraction becomes evident with emerging intercellular cracks and slightly folded cell walls, accompanied by a C/O ratio increase to 1.31. This drying stage shows minimal volumetric reduction and no significant chromatic change, indicating that the cellular integrity is largely maintained during 2.70 min. Extended drying to 3.66 min (Figure 14c) induces intensified cellular shrinkage, a notable proliferation of surface porosity, and moderate cell wall contraction, yielding a C/O ratio of 1.42. The specimen develops light yellow pigmentation with uniform volumetric shrinkage. This stage reflects a more pronounced impact of dehydration on the microstructure, where the cellular components begin to exhibit visible signs of stress and reorganization. Upon reaching 4.30 min (Figure 14d), complete structural collapse occurs in multiple regions, forming a network-like morphology with enlarged pores and a peak C/O ratio of a 1.65. Distinct caramelized yellow coloration emerges along the specimen edges alongside pronounced volumetric reduction [52]. This drying stage highlights the extreme effects of prolonged microwave exposure, leading to significant changes in both the physical and chemical properties of the TM. EDS analysis confirms C, O, N, and K as primary constituents of TM. The progressive C/O ratio elevation correlates with drying time, reflecting water loss-induced elemental concentration. Cellular wall regions exhibit accumulated elemental deposition, ultimately shaping the porous network structure through continuous dehydration. These morphological transitions align with observed macroscopic changes: gradual volumetric contraction and progressive color darkening from pale cream to golden yellow.

#### 3.4.4. Enhanced Dehydration Mechanism of Microwave-Dried TM

**Figure 15:**
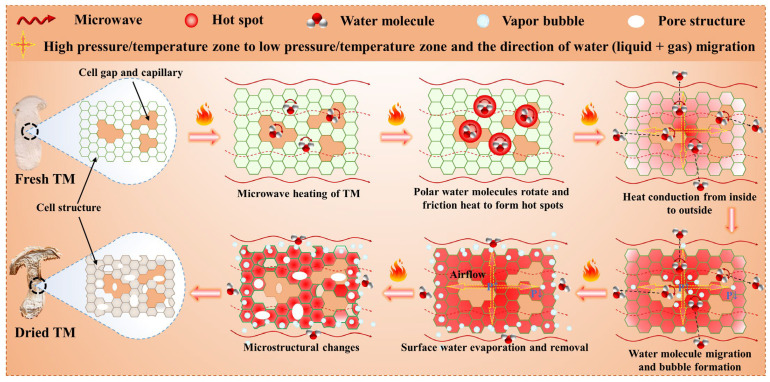
Schematic diagram illustrating the enhanced dehydration mechanism of microwave-dried TM.

Figure 15 presents a schematic diagram illustrating the enhanced dehydration mechanism during MD of TM. Microwave energy penetrates the TM tissue, and its alternating electromagnetic field induces rapid rotation and frictional heating of internal polar water molecules, thereby converting electromagnetic energy directly into thermal energy. This process enables rapid volumetric heating, leading to the rapid vaporization of internal moisture and its accumulation within the enclosed cellular or pore structures, forming a high-pressure zone (P↑). The resulting pressure gradient between this internal high-pressure zone and the external low-pressure drying environment (P↓) serves as the primary driving force for water migration from the interior to the surface of the material [53]. Under the influence of this pressure gradient, both liquid water and steam are forced through the pore network to the surface, where they evaporate and are removed, thereby maintaining the pressure differential and continuously promoting the dehydration process. The structure of the TM material also evolves during drying. The rupture or deformation of cell walls due to high temperatures can alter the pathways for water migration and enhance the rate of dehydration. Moreover, the porosity of the material may increase, facilitating the rapid migration and evaporation of water. While MD primarily relies on volumetric heating, heat conduction and convection also contribute to the overall drying process. Heat conduction helps in maintaining a uniform temperature distribution within the material, while convection aids in the removal of water vapor from the surface, maintaining the pressure differential and driving the dehydration process. In contrast to conventional drying methods, MD generates internal pressure through volumetric heating, facilitates water transport via pressure-driven flow, and achieves surface evaporation and removal. This mechanism is fundamentally different from that of traditional hot-air drying, which relies on external heat conduction, surface evaporation, and slow internal moisture diffusion [54]. In summary, MD of TM, characterized by its distinct heating mechanism and water transport dynamics, offers notable advantages in terms of drying rate, energy efficiency, and product quality preservation.

#### 3.4.5. Economic Benefit Analysis

A techno-economic model was established to compare the variable drying cost (C_dry_, USD/kg water removed) between the proposed microwave process (MW) and a conventional hot-air (HA) tunnel dryer. The model considers electricity price (E_p_ = 0.12 USD/kWh; China industrial tariff, 2024), labor wage (L_w_ = 2.5 USD/h), and annualized capital charge (ACC). ACC for the 6 kW pilot-scale microwave unit was calculated as follows:

(5) ACC = (CAPEX × CRF)/Q_a_

where CAPEX = 18,000 USD, CRF (capital recovery factor) = 0.117 (10 yr life, 8% discount rate), and annual throughput Q_a_ = 110 t water/y (based on 15 kg/h feed × 7500 h/y). Under the optimized operating point (Power = 620.00, Time = 2.63 min), specific electricity consumption was 27.18 kWh/t water (75 °C, 0.8 m/s), being significantly lower than the 32.83 W·h under the power of 500.00 W at 3.94 min. Consequently, MW reduces the energy-related variable cost by 17.2%. Including labor and ACC, the total C_dry_ is 0.021 USD/kg water for MW versus 0.034 USD/kg water for HA, yielding a 38% overall cost reduction. These results align with the intermittent-power microwave model proposed by Teleken et al. [50], which predicted ≥15% energy savings when high power pulses. The mechanism involves high-power, short-time drying generating significant vapor pressure gradients. This “vapor pressure-driven” flow facilitates rapid internal moisture migration towards the surface, reducing reliance on slower diffusion and surface evaporation. This mechanism minimizes thermal degradation, helping preserve nutrients, flavor, and color. Consistent with SEM observations, TM dried under optimized MW conditions exhibited superior rehydration properties and maintained tissue integrity, benefiting texture and overall quality. Furthermore, the significantly shortened drying cycle enhances processing capacity per unit time, offering considerable economic advantages.

### 3.5. Pilot-Scale MD Experiment of TM

**Figure 16:**
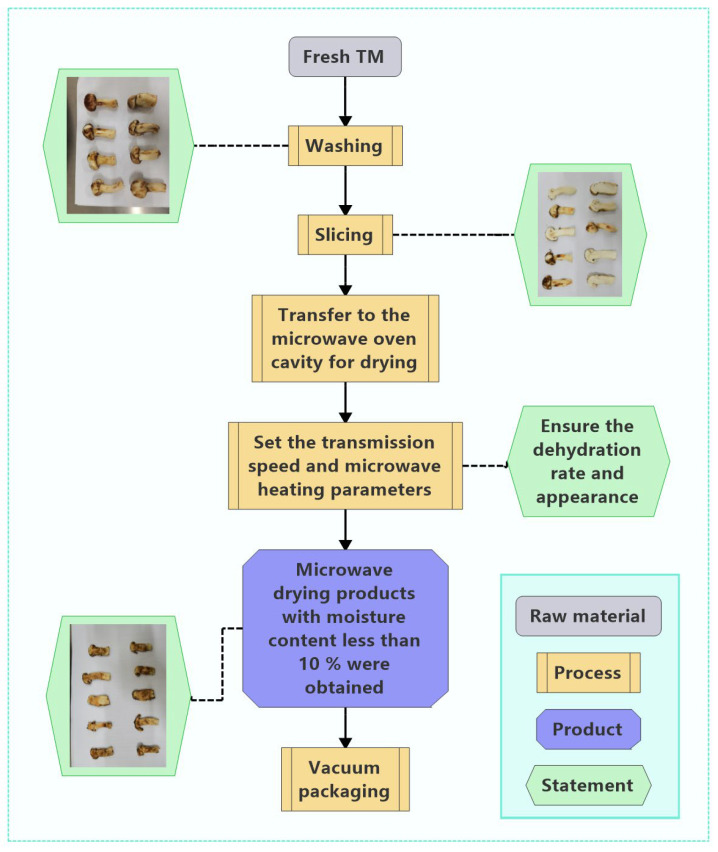
Pilot-scale process flow diagram for MD of TM.

**Figure 17:**
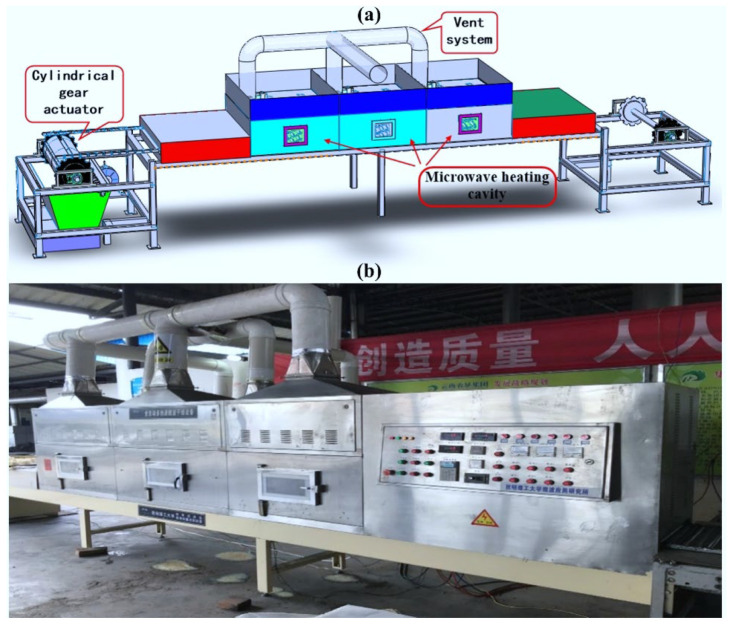
The schematic diagram of TM pilot process, (**a**) Schematic configuration of the pilot-scale MD system for TM; (**b**) Photographic documentation of the pilot-scale MD system for TM.

Laboratory-scale results confirm the efficacy of MD for fresh TM. Based on these findings, Figure 16 presents a scaled industrial process. The workflow initiates with cleaning and slicing, followed by continuous conveying into MD equipment (Figure 17). Optimized power-time parameters achieve the target DR while preserving morphological integrity, yielding products with ≤10% moisture content (validated in Section 3.4.3). Final products undergo vacuum sealing prior to distribution. To ensure consistent scale-up from the 620.00 W batch system (laboratory) to the 6.00 kW continuous belt apparatus (pilot), the power per unit mass was maintained at 0.40 kW/kg (laboratory: 0.62 kW/1.5 kg = 0.41 kW/kg; pilot: 6 kW/(15 kg/h × 2.63/60 h) = 0.41 kW/kg), following the energy similarity principle proposed by Radoiu et al. [55]. The pilot dryer (Model MWP-6, Nanjing Sanle) features were as follows: 2.45 GHz multimode cavity (1200 × 400 × 300 mm); PTFE-coated glass-fiber conveyor belt (corrected speed: 0.46 m/min); three magnetrons (2.0 kW each) in staggered array (±5% field uniformity); Inline IR camera (FLIR A700sc) for temperature mapping; and load cell (0–30 kg, ±2 g) for mass monitoring. Under optimized conditions (6 kW power, 0.46 m/min belt speed, 2.63 min residence time), the system achieved a feed rate of 15.0 ± 0.2 kg/h fresh TM (87% moisture), outlet moisture of 9.8 ± 0.3% w.b., and specific energy consumption: 1.86 MJ/kg water (vs. 1.74 MJ/kg lab-scale, +6.9% deviation). The pilot system reached 88% of laboratory capacity (17.1 kg/h equivalent lab throughput vs. 15 kg/h pilot), demonstrating robust scalability for industrial deployment [56].

## 4. Conclusions

This study systematically explored the application potential and underlying mechanisms of MD technology in the dehydration process of the rare edible TM. The research clarified the three-stage dehydration pathway of TM material (free water, weakly bound water, and bound water), with the rapid evaporation of free water (accounting for 69.8% of the total weight loss) being the key to efficient drying. This pyrolysis process is closely coupled with dielectric properties: the changes in the ${\varepsilon_{r}}^{\prime }$ and ${\varepsilon_{r}}^{\prime \prime }$ are directly related to the state of moisture, confirming that dipole loss is the main heating mechanism. The tanδ increased instead of decreasing at high temperatures (by 213.0%), indicating that in the later stages of drying, the enhanced conductive losses due to carbonization of the material also promote heating, providing a theoretical basis for adopting high-power, short-time treatment strategies. A high-precision (R^2^ = 0.97) quadratic polynomial model was constructed using RSM, finding that microwave power is the most critical factor affecting drying rate, followed by drying time and material quantity, and the optimal process parameters were determined (power: 620.00 W, time: 2.70 min, mass: 13.20 g). Under these conditions, an ideal DR of 85.41% can be stably achieved. Combined with SEM microstructure analysis, it was found that 2.70 min is the key balance point between maintaining cell structural integrity and achieving efficient dehydration, avoiding quality degradation caused by over-drying. The volumetric heating effect of microwaves generates a huge vapor pressure inside TM, forcing moisture to migrate outward, fundamentally overcoming the conduction and diffusion limitations of traditional drying methods and achieving efficient short-time heating. Economic model analysis showed that compared to traditional HA drying, the optimized microwave process can reduce total costs by 38%. Finally, a successful 20-fold pilot-scale experiment verified the robustness and scalability of the technical solution. In conclusion, this study provides a verified technological pathway for the high-value, efficient, low-consumption, and high-quality processing of TM and similar edible fungi, and the proposed dielectric loss mechanism and moisture migration mechanism deepen the scientific understanding of MD of food. However, the study focused only on TM, limiting the generalizability of the findings. Future research should test the proposed MD technology on a wider range of fungi and food products to better assess its adaptability.

## Figures and Tables

**Table 1:** Comparison of MD with other drying methods for food processing.

Method	Material	Key Findings	Source
MD, hot air, vacuum freeze drying	Lotus flower tea	400 W MD demonstrated high efficiency with effective preservation of phenolic compounds in lotus flower tea, resulting in enhanced antioxidant activity.	[16]
Hot air, MD, hybrid hot air–MD	Cabbage	MD samples retained 67.3% vitamin C, 90.0% free phenols, and 86.6% total phenols, demonstrating stronger antioxidant activity.	[17]
Hot air, freeze, MD	Carrot	MD products exhibited optimal rehydration capacity and high α-carotene and vitamin C levels, with sensory attributes comparable to freeze-dried samples.	[18]
Vacuum freeze, MD, hybrid vacuum freeze–hot air	Shiitake mushroom slices	MD samples showed the lowest formaldehyde content.	[19]
Shade, sun, hot air, vacuum freeze, MD	Safflower	MD-treated samples contained higher hydroxysafflor yellow A, kaempferol, and total flavonoids.	
MD, freeze, hot air convection, vacuum drying	Red chili powder	MD required the shortest drying time, yielding products with brighter color, higher particle density, and lower porosity, enhancing storage stability.	[20]
Hot air, vacuum, vacuum freeze, MD	Artichoke powder	MD achieved the fastest drying rate, producing powders with maximum bulk density, optimal water/oil holding capacities, and highest crude fat content.	[21]

**Table 2:** Calculation of wet basis moisture content in TM.

Sample Name	Trial 1	Trial 2	Trial 3	Average Moisture Content
TM	86.64%	90.51%	88.25%	88.47%

**Table 3:** Response surface design test level and coding of factors.

Factors	Codes	Levels				
		−1.682	−1	0	1	1.682
Power/W	A	298.19	380.00	500.00	620.00	701.82
Time/min	B	2.03	2.63	3.51	4.38	4.98
Mass/g	C	6.95	9.82	14.03	18.23	21.09

**Table 4:** Experimental results of response surface regression model optimization.

Run	Factor 1	Factor 2	Factor 3	Response Value
	A: Power /W	B: Time /min	C: Mass /g	DR /%
1	380.00	2.63	9.82	63.72
2	620.00	2.63	9.82	85.42
3	380.00	4.38	9.82	75.84
4	620.00	4.38	9.82	91.29
5	380.00	2.63	18.23	51.38
6	620.00	2.63	18.23	80.30
7	380.00	4.38	18.23	65.85
8	620.00	4.38	18.23	88.24
9	298.19	3.51	14.03	52.89
10	701.82	3.51	14.03	89.82
11	500.00	2.03	14.03	75.32
12	500.00	4.98	14.03	86.78
13	500.00	3.51	6.95	89.71
14	500.00	3.51	21.09	80.17
15	500.00	3.51	14.03	86.55
16	500.00	3.51	14.03	83.44
17	500.00	3.51	14.03	85.14
18	500.00	3.51	14.03	83.25
19	500.00	3.51	14.03	84.79
20	500.00	3.51	14.03	82.44

**Table 5:** ANOVA results for the response surface quadratic regression model.

Source	Sum of Squares	df	Mean Square	F-Value	*p*-Value (Prob > F)	Significance
Model	2561.46	9	284.61	44.73	<0.0001	Significant
A–Power	1660.04	1	1660.04	260.90	<0.0001	
B–Time	260.74	1	260.74	40.98	<0.0001	
C–Mass	158.63	1	158.63	24.93	0.0005	
AB	20.42	1	20.42	3.21	0.1035	
AC	25.06	1	25.06	3.94	0.0752	
BC	2.44	1	2.44	0.38	0.5494	
A^2^	406.98	1	406.98	63.96	0.00001	
B^2^	51.28	1	51.28	8.06	0.0175	
C^2^	3.76	1	3.76	0.59	0.4595	
Residual	63.63	10	6.36			
Lack of fit	52.32	5	10.46	4.63	0.0590	Not significant
Pure error	11.30	5	2.26			
Cor total	2625.09	19				

df—Degrees of freedom; $R^{2}$ = 0.97; ${R_{adj}}^{2}$ = 0.95; C.V.% = 3.19; *Adeq Precision* = 22.84; standard deviation = 2.52; *p*-value < 0.05 indicates that model terms are significant.

**Table 6:** Verification of predicted value of quadratic regression response model.

Power/W	Time/min	Mass/g	Predicted DR/%	Measured DR/%	Absolute Deviation/%
620.00	0.00	13.20	0.00	0.00	0.00
620.00	2.70	13.20	83.50–85.50	85.41	≤1.50
620.00	3.66	13.20	85.50–87.50	86.90	≤1.50
620.00	4.30	13.20	88.00–89.50	89.15	≤1.50

## Data Availability

The original contributions presented in the study are included in the article, further inquiries can be directed to the corresponding authors.

## Abbreviations

The following abbreviations are used in this manuscript:

TM	Tricholoma matsutake
MD	Microwave drying
RSM	Response surface methodology
DR	Dehydration rate
CCD	Central Composite Design
ε r′	Relative permittivity
ε r″	Relative dielectric loss
tan δ	Loss tangent
TG	Thermogravimetric
SEM	Scanning electron microscope
EDS	Energy-dispersive X-ray spectroscopy
DSC	Differential scanning calorimetry
